# Automated Procedure for Contact-Map-Based Protein Structure Reconstruction

**DOI:** 10.1007/s00232-014-9648-x

**Published:** 2014-03-29

**Authors:** Bogumil M. Konopka, Marika Ciombor, Monika Kurczynska, Malgorzata Kotulska

**Affiliations:** Institute of Biomedical Engineering and Instrumentation, Wroclaw University of Technology, Wybrzeze Wyspianskiego 27, 50-370 Wrocław, Poland

**Keywords:** Ion channel, Protein structure, Contact-maps, Protein structure reconstruction, Protein electrostatics

## Abstract

Knowledge of the three-dimensional structures of ion channels allows for modeling their conductivity characteristics using biophysical models and can lead to discovering their cellular functionality. Recent studies show that quality of structure predictions can be significantly improved using protein contact site information. Therefore, a number of procedures for protein structure prediction based on their contact-map have been proposed. Their comparison is difficult due to different methodologies used for validation. In this work, a Contact Map-to-Structure pipeline (*C2S_pipeline*) for contact-based protein structure reconstruction is designed and validated. The *C2S_pipeline* can be used to reconstruct monomeric and multimeric proteins. The median RMSD of structures obtained during validation on a representative set of protein structures, equaled 5.27 Å, and the best structure was reconstructed with RMSD of 1.59 Å. The validation is followed by a detailed case study on the KcsA ion channel. Models of KcsA are reconstructed based on different portions of contact site information. Structural feature analysis of acquired KcsA models is supported by a thorough analysis of electrostatic potential distributions inside the channels. The study shows that electrostatic parameters are correlated with structural quality of models. Therefore, they can be used to discriminate between high and low quality structures. We show that 30 % of contact information is needed to obtain accurate structures of KcsA, if contacts are selected randomly. This number increases to 70 % in case of erroneous maps in which the remaining contacts or non-contacts are changed to the opposite. Furthermore, the study reveals that local reconstruction accuracy is correlated with the number of contacts in which amino acid are involved. This results in higher reconstruction accuracy in the structure core than peripheral regions.

## Introduction

Knowledge of the three-dimensional structure of a protein is one of the key elements toward understanding the molecular mechanisms that underlie protein function. Currently, only 2,061 transmembrane protein structures are known (PDBTM, as of 31.01.2014, Kozma et al. 2012), while in the Protein Data Bank (PDB, as of 26.11.2013, Berman et al. 2000) 88,725 protein structures are deposited. Evaluations of computational methods for protein structure prediction, carried out during biannual CASP contests (Critical Assessment of Techniques for Protein Structure Prediction), show that significant progress has been made in the field since the contests began (Kryshtafovych et al. 2013). Homology modeling methods can deliver fine structure predictions, if structural templates are available (Söding et al. 2005; Arnold et al. 2006; Kelley and Sternberg 2009; Källberg et al. 2012). For instance, Memoir (Ebejer et al. 2013) a program, which was specially designed to predict membrane proteins, provides models with average Root Mean Square Deviation (RMSD) of 2.57 Å. Prediction of transmembrane structures, especially ionic channels, will further allow for modeling their conductivity characteristics using biophysical models (e.g., Dyrka et al. 2008; Dyrka et al. 2013) and finally prediction of their cellular functionality (e.g., Jafri and Kotulska 2006). Currently, the major challenge in this field is to predict the protein structure, without prior knowledge of homologous structures. Recent studies show that using protein contact site information can significantly improve the quality of de novo structure predictions (Nugent and Jones 2012; Hopf et al. 2012).

A protein contact site, also called a residue–residue contact, is a pair of amino acid residues located within a certain distance threshold of one another (Duarte et al. 2010a). A set of contact sites, defined for a protein, constitutes a contact-map. The most recently published report from the CASP evaluation of residue–residue contact site predictors concluded that the performance of state-of-the-art methods was not satisfactory (average contact site prediction accuracy equaled 16.8 % (Monastyrskyy et al. 2011)). However, the work of Marks et al. (Marks et al. 2011) and Jones et al. (Jones et al. 2012) showed that the approach using evolutionary sequence variation could yield very accurate contact site prediction. If we are able to predict enough amino acid contacts, then it would be possible to reconstruct the whole protein structure. The question arises how many protein contacts need to be predicted and what is the quality of proteins built based on such residue–residue interactions.

So far a number of studies have been conducted that proposed and validated procedures for contact-map-based protein structure prediction. In (Duarte et al. 2010a, b) and (Marks et al. 2011), a well-established algorithm for NMR structure determination was used (Havel et al. 1983), followed by simulated annealing structure refinement. In (Vendruscolo et al. 1997) a heuristic method of growing the amino acid chain of monomers one by one was proposed. The growth process was guided by a contact-based cost function and followed by a structure adaptation stage, which accepted changes in the structure using the Metropolis criterion. Vassura et al. proposed a heuristic method that perturbs the coordinates of Cα carbons in order to produce a structure with a contact-map as close as possible to the input contact map. The studies report structure accuracies in the range of 1.5–4.5 Å. However, these values cannot be compared due to different methodologies used for validation. The studies differ in terms of protein test sets and structure quality measures. For instance, in (Duarte et al. 2010a; Vassura et al. 2008) validations were limited to reconstruction of protein Cα traces. The studies in (Hopf et al. 2012) and (Nugent and Jones 2012) were limited to prediction of transmembrane proteins, while those in (Taylor et al. 2012), and (Marks et al. 2011) were limited to evaluation on a set of several globular folds. In order to clearly and comprehensively estimate the potential of predicting protein structures based on contact maps, a validation on a representative set of protein structures with several measures of structure quality should be performed. In this work, an automated Contact Map-to-Structure pipeline (*C2S_pipeline*) for contact-based protein structure reconstruction, using available bioinformatics tools, is presented and validated. The pipeline can be used to reconstruct proteins consisting of single amino acid chains, as well as multimeric proteins. We present a two-step validation of the pipeline. First the validation is performed on a representative set of protein structures, and then a detailed case study on the KcsA ion channel is performed.

## Methods

### The *C2S_pipeline* for Single Chain Protein Reconstruction

**Fig. 1:**
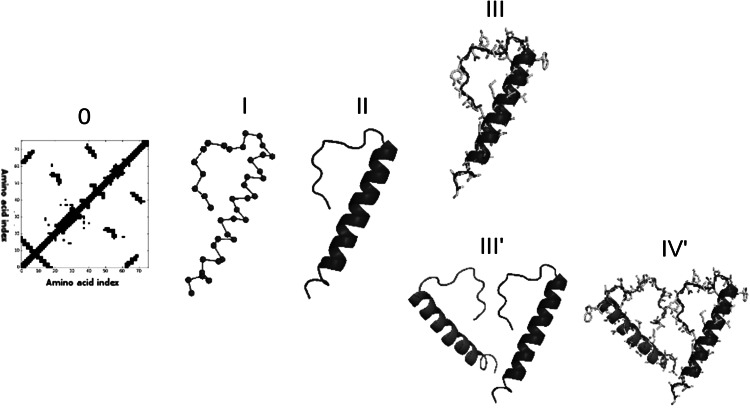
The contact map-based protein reconstruction procedure is performed in a sequence of steps: (I) FT-COMAR is used to acquire the Cα trace of the structure based on the contact map, (II) SABBAC is used to reconstruct the protein backbone, (III) and (IV′) SCWRL is used to add amino acid side chains and optimize the structure geometry, (III′) in case of ion channel structures subunit symmetric projection is performed. A contact map (0) is the input information which is fed into the pipeline

The pipeline takes as an input a protein Contact Map (CMAP). Reconstruction of single chain proteins is performed in a three step protocol (Fig. 1): (1) C-alpha trace reconstruction with FT-COMAR (Vassura et al. 2008); (2) backbone reconstruction with SABBAC (Maupetit et al. 2006), (3) side-chain prediction and structure optimization with SCWRL (Krivov et al. 2009). Each step is described in greater detail below. The protocol outputs a full-atom 3D structure of a protein.

#### Reconstruction of the C-alpha Trace (Fig. 1(I))

FT-COMAR is used to determine location of the C-alpha atoms (Vassura et al. 2008). It is based on spatial restrains imposed by residue–residue contacts and it treats atoms as geometrical points in three dimensional space. Importantly, the information on the amino acid sequence of the protein is not used in the process. The algorithm can be divided into two separate phases. In the first phase, a partially random structure is generated. In the second phase, the structure is perturbed and refined in order to satisfy the restraints induced by the input contact matrix. The program assumes that consecutive amino acids in the input CMAP are connected with the peptide bond; therefore, they should be in close proximity to one another in the 3D space. The algorithm tries to apply this assumption and holds neighboring residues together.

#### Reconstruction of the Protein Backbone (Fig. 1(II))

Atoms forming the main chain are rebuilt with SABBAC, an application that allows for a rapid reconstruction of the main chain of a protein based on C-alpha coordinates. SABBAC was designed to reconstruct single polypeptide chains. The method uses a library of structural fragments four amino acid long, the so called Structural Alphabet (SA) (Camproux et al. 2004), which are assembled together. The SA was built using Hidden Markov Model framework (Camproux et al. 2004). A “greedy algorithm” is used to get an optimal combination of fragments (Tuffery et al. 2005), (Maupetit et al. 2006), which is compatible with the input C-alpha trace.

#### Addition of Side-Chains and Structure Optimization (Fig. 1(III))

The final phase of the structure reconstruction, addition of amino acid side chains and rotamer optimization, is performed using SCWRL4 (Krivov et al. 2009), which is a well-established tool for solving the side chain prediction problem. SCWRL uses a backbone dependent rotamer library to get a first approximation of the side-chain coordinates. Then it calculates energies and constructs an interaction graph in which vertices denote amino acid residues and edges are interactions (Krivov et al. 2009). It optimizes the arrangement of particular rotamers by a graph decomposition and energy minimization methods. SCWRL outputs the coordinates of the final, full-atom model structures.

### Reconstruction of Multimeric Proteins with *C2S_pipeline*

FT-COMAR and SABBAC, which are used in the single chain protein reconstruction pipeline, were not designed to cope with multi-chain proteins. FT-COMAR assumes that consecutive residues in the input CMAP are close together in the three-dimensional space. This assumption is correct in case of monomeric proteins since all residues belong to the same amino acid chain. However in case of multimeric proteins, terminal amino acids of different chains may be described in consecutive rows of the CMAP despite being distant from one another in the 3D space.

In order to adapt the monomeric protein reconstruction pipeline to reconstruction of multimeric proteins, two additional steps were introduced. The procedure is the following: (*1) Dummy amino acid loops insertion*, (2) C-alpha trace reconstruction with FT-COMAR (Vassura et al. 2008), (3) backbone reconstruction with SABBAC separately for each chain (Maupetit et al. 2006), (*4) symmetry-based assembly of protein subunits*, and (5) side-chain prediction and structure optimization with SCWRL (Krivov et al. 2009).

#### Dummy Amino Acid Loops

In a CMAP of a multimeric protein, the sequences of all chains are concatenated. Therefore, the CMAP holds information about all contacts sites of the protein (intra and inter-chain). For example, in a homodimer of two 100–residue long subunits, the residue indexed as 100 is the C-terminus of chain A, and residue indexed as 101 is the N–terminus of chain B. The actual geometrical distance between the two amino acids can be high despite the fact that the residues are “neighbors” in the contact matrix. FT-COMAR keeps the terminal amino acids close in 3D space, which results in deterioration of reconstruction quality.

In order to improve the reconstruction quality for multimeric proteins, such as ion channels, we insert artificial loops of dummy amino acids into the CMAP between chain terminals prior running FT-COMAR. These loops are trimmed from the structure after reconstruction. The authors of FT-COMAR showed that introducing of non-existing contacts into the contact map has a strong negative effect on the reconstruction quality, thus they proposed to mark some inter-residue contacts as uncertain by “-1” in the contact matrix. These contacts are not taken into account during structure reconstruction. We use this notation while inserting artificial loops. Each dummy atom that forms the inserted loop is an additional row and additional column of −1 in the contact matrix. The loop is neutral to the reconstruction process while moving the neighboring terminal residues of different chains away from each other, which improves the performance of FT-COMAR.

#### Symmetry-Based Assembly of Protein Subunits

In case of ion channels, the backbone reconstruction step [“Reconstruction of the Protein Backbone (Fig.1.II)” section] is carried out separately for each chain of the protein. Therefore, this step returns four backbones. Each of the backbones is then used to rebuild a whole ion channel on the basis of the channel axis symmetry. First, the chains are reassembled to form an asymmetric channel. The structure is positioned so that the axis of the pore lies on the z axis. This is done by i) aligning the channel to a similar-sized structure from the Orientations of Proteins in Membranes (OPM) database (Lomize et al. 2006) to get the proper channel axis direction, and then by ii) moving the protein to the beginning of the reference by a translation. After that each of the subunits is projected 4 times using the axis of symmetry to form a full tetrameric channel. This procedure produces 4 symmetric ion channels. Additional ion channel structure is produced by projecting the averaged subunit.

### Validation on a Set of SCOP-ASTRAL Domains

The validation set was built following the guidelines provided by Söding (Söding et al. 2005). 1961 representatives of SCOP superfamilies, as supplied by SCOP on-line interface, were downloaded (http://astral.berkeley.edu/scopseq-1.75.html as of 20.09.2012). Due to time limitations caused by the availability of the SABBAC server, 205 structures were used in the final validation set. In terms of SCOP-ASTRAL classes (Table 1) most of the structures belonged to *all-alpha*, *all-beta*, and *alpha* *+* *beta* proteins. About 10 % of structures of these three classes were used. It is important to note that the *multidomain* and *membrane* classes had only a few representatives in the validation set, which covered only 4–6 % of structure of these classes. On the other hand, the number of domains from the *small protein* class was 25 which consisted about 20 % of *small proteins* class.

**Table 1:** The validation set comprised structurally diverse proteins from different SCOP-ASTRAL structures

SCOP-ASTRAL class	Structure numbers in SCOP database	Structure numbers was used in the validation	Percent of the SCOP structure was used in the validation
*all-alpha*	507	55	10.8 %
*all-beta*	354	41	11.6 %
*alpha/beta*	244	24	9.8 %
*alpha* *+* *beta*	552	50	9.1 %
*multidomain*	66	3	4.5 %
*membrane*	109	7	6.4 %
*small proteins*	129	25	19.4 %

The validation was carried out as follows. First, a contact map (CMAP) was derived from a native structure with the use of PconPy (Ho et al. 2008). The map was next used in the *C2S_pipeline*. The resulting full-atom structure was compared to the native structure with a full atom RMSD. For every analyzed protein, 50 model-structures were generated and their RMSD value averaged. The relationships between RMSD, sequence length, and contact density (CD, defined as the average number of residue–residue contacts formed by amino acid) were analyzed. Formally CD is defined as:

$$CD = \frac{1}{L}\sum\nolimits_{i = 1}^{L} {c_{i} },$$

where *L* is protein sequence length, *c*_*i*_ is the number of contacts in which the *i-th* amino acid participates.

### Case Study Validation–KcsA Ion Channel

Based on experimentally solved native structure of the KcsA channel—3fb8 in the PDB database (Berman et al. 2000), a CMAP was created. Several testing experiments were conducted. Structural models were reconstructed based on:

1. complete contact map (complete-CMAP structures);
2. contact map with information regarding positive contacts (no information regarding non-contacts, positive-only-CMAP structures);
3. contact maps with the numbers of contacts and non-contacts reduced to 90, 70, 50, and 30 %, (reduced-CMAP structures). The status of remaining contacts and non-contacts was assumed as unknown (“-1”);
4. erroneous contact maps with 90, 70, 50, and 30 % of correct contacts in which the remaining contacts and non-contacts were changed to opposite (erroneous-CMAP structures);

Contact map reduction in point (3) was conducted by substitution of randomly chosen contacts (‘1’ in the CMAP) and non-contacts (‘0’ in the CMAP) with ‘-1’. In point (4) random ‘1’ were selected and changed to ‘0’. In both cases, the reduction was repeated 10 times, and 50 structural models were generated based on each randomized CMAP.

**Fig. 2:**
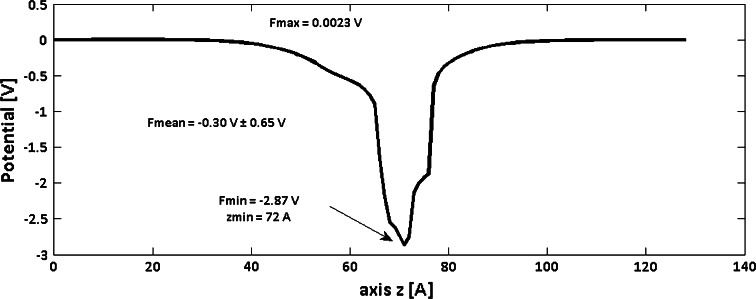
The electrostatic potential profiles along the channel axis (*z*) of the KcsA PDB-structure. The profile features were shown: the maximal potential (*Fmax*), the minimal potential (*Fmin)*, the position of the minimal potential (*zmin*), and average potential with standard deviation (*Fmean*)

For each model-structure a number of structural parameters were calculated:

1. global full-atom RMSD, full-atom alignment;
2. selectivity filter full-atom RMSD, selectivity filter alignment;
3. RMSD of particular residues at full-atom alignment (used for Local RMSD calculations);

In addition, in order to investigate to what extent contact maps of ion channels can be reduced without causing a loss of functionality, the distributions of the electrostatic potential inside the channel were calculated. The electrostatic potential was calculated with the Adaptive Poisson-Bolzmann Slover (APBS) (Baker et al. 2001). The simulation box was a cubic 129 × 129 × 129 with grid space of 1 Å. The Poisson-Bolzmann equation was solved with membrane potential equal to 0 V without ions in solution. The dielectric constant of the protein was *ε* = 4. Elsewhere, including the inside of the pore, it was equal to the dielectric constant of the electrolyte, *ε* = 80. All electrostatic potential profiles were compared to the template profile (Fig. 2), which was obtained for the native structure of the KcsA potassium channel. The profiles were parameterized with four measures: the maximum value of the potential profile (*Fmax*), the minimum value of the potential profile (*Fmin)*, the position of the minimum potential (*zmin*), and the root mean squared error (RMSE) of the profile compared to the template. Since RMSD measures the differences between structures, we used relative differences between *Fmax*, *Fmin* and *zmin* of evaluated models and the template to describe the relationship between structural and electrostatic quality of the models.

## Results

The reconstruction protocol was tested in two assessment studies. First, a validation on a large set of structurally diverse structures was carried out. The main objective was to estimate the potential of the procedure to generate high-quality structures. This was followed by a case-study of a single protein structure reconstruction. The purpose of the second study was to investigate how external factors, such as contact map completeness or error rate, influence the prediction accuracy.

### Validation on a Set of SCOP-ASTRAL Domains

**Fig. 3:**
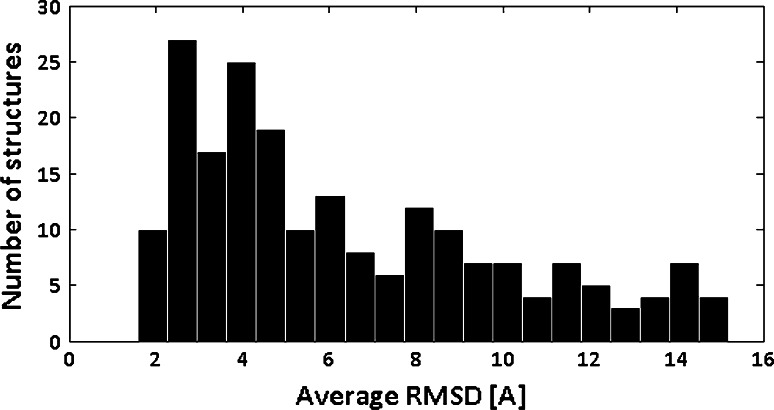
The distribution of the average RMSD acquired for studied protein structures

The average RMSD values (Fig. 3) acquired for majority of proteins were below 5 Å, with the overall distribution median of 5.27 Å. The average RMSD distribution was positively skewed, so the number of structures with low RMSD was higher than the structures with high RMSD. The average RMSDs were accumulated around mean value as confirmed by kurtosis of 2.50. The best RMSD, 1.59 Å, was acquired for the 82-amino acid long, *all-beta* protein. Contact density of the protein equaled 13. The worst case was the 205 amino acid long protein from the *alpha/beta* SCOP-ASTRAL class. The protein density was 10.98, and the acquired average RMSD was over 15 Å.

**Fig. 4:**
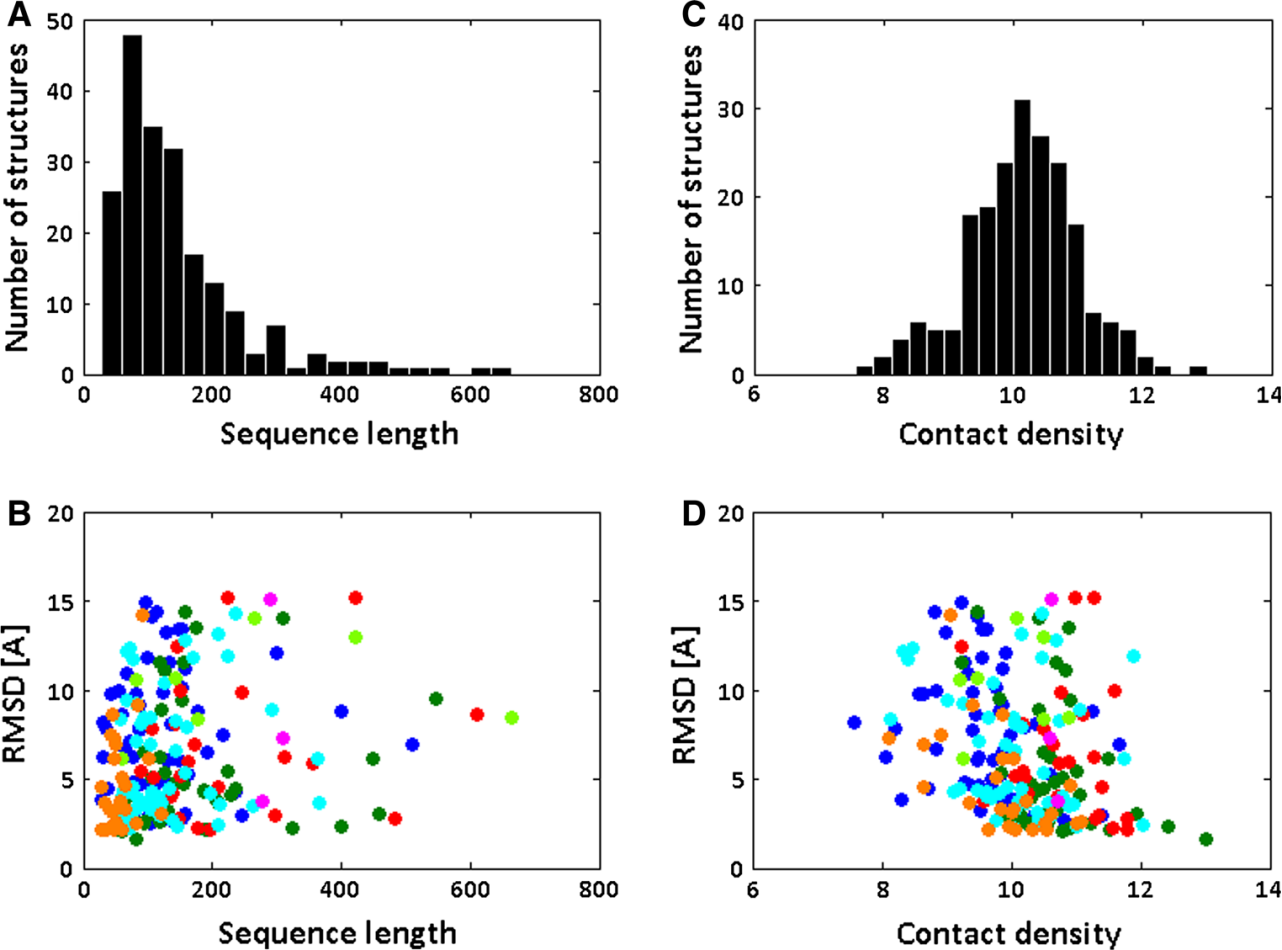
The relation between the average RMSD values and the protein. (**a**) The distribution of the sequence length for tested protein structures. (**b**) The scatterplot of the average RMSD values and the protein sequence lengths. (**c**) The distributions of the structure contact density. (**d**) The scatterplot of the average RMSD values and the protein contact density. The *circles* color depend on the SCOP-ASTRAL classes: *all-alpha* (*blue*), *all-beta* (*dark green*), *alpha/beta* (*red*), *alpha* *+* *beta* (*cyan*), *multidomain* (*magenta*), *membrane* (*bright green*), and* small proteins* (*orange*). The set consisted of 205 protein domains downloaded from SCOP-ASTRAL database (Color figure online)

The distribution of sequence length is depicted in Fig. 4a. The average protein length was 148, however the distribution was positive-skewed (skewness 2.03) and strongly leptokurtic (kurtosis 7.80) so several much longer proteins were present in the set. It is not possible to unambiguously state whether protein length influences prediction accuracy, neither in the whole set, the *τ* Kendall correlation was 0.13 (for α-level 0.05), nor for each of the SCOP-ASTRAL class separately (Fig. 4b). Also, it should be noted that there were only 18 proteins of a length greater than 300, and this result may not be representative enough to draw general conclusions.

Structures were also characterized in terms of structure contact density (Fig. 4c). The distribution of the contact density was condensed around the median value 10.18 that was certified by kurtosis value 3.48. The average value of contact density 10.15 was similar to the median value and the skewness equaled −0.08, so the distribution of contact density was almost symmetric. The contact density of proteins is related to the quality of models, the *τ* Kendall correlation was −0.23 (Fig. 4d). The accuracy of a model-structures acquired for more densely packed structures was greater. The highest correlation between the average RMSDs and the contact density was observed in *small protein* class: *τ* Kendall correlation was −0.41. The values of the contact density in this class were between 8.11 and 11.08, with the median of 9.96. Nevertheless, a significant decrease of RMSD values was observed for proteins with more than 10 contacts per amino acid. The hypothesis of equal medians for proteins: with more than 10 contacts per amino acid and with less than 10 contacts per amino acid was rejected using the Wilcoxon rank sum test at 5 % significance level (Z-value was 4.77 and *p*-value of 1.85 × 10^−6^). This could be explained by the fact that the most important component of the RMSD depends on the stage of FT-COMAR reconstruction, which is based on geometrical restraints imposed by the contact map. The more contacts an amino acid creates the more precisely its 3D localization can be estimated.

**Fig. 5:**
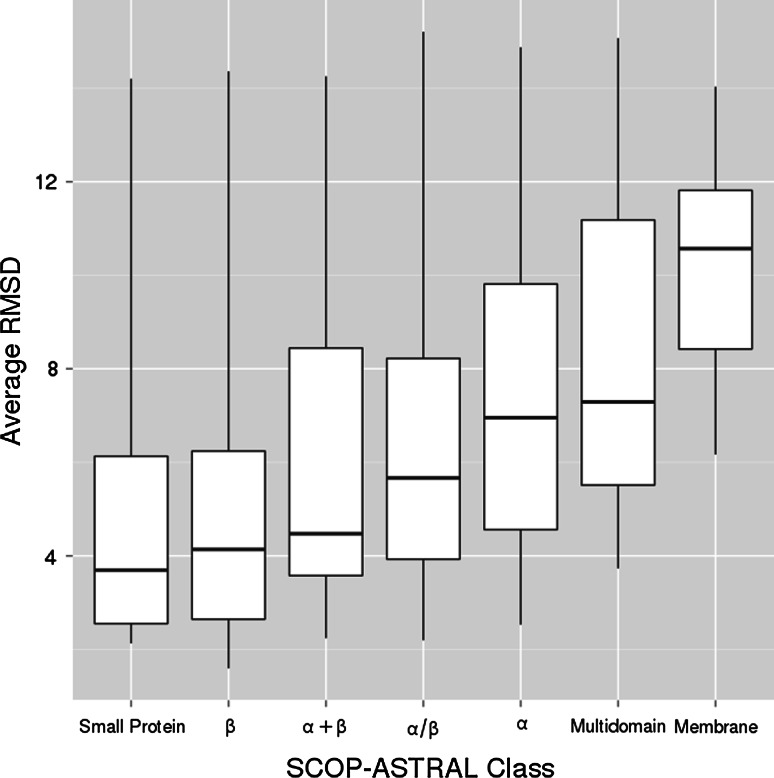
Boxplots of the average RMSD values in proteins from different SCOP-ASTRAL classes. Box borders denote QI and QIII quintiles, the median is marked with the *thick line*. Box whiskers denote maximal and minimal values

Finally, we tested whether any classes of proteins are reconstructed with better accuracy. The validation shows that the protocol was the most successful in case of *small* and *all-beta* proteins (Fig. 5). The quality of the structure models of *small* and *all-beta* proteins was statistically significantly better than *all-alpha* and *membrane* proteins, which was tested with the multicompare Kruskal-Wallis test at 5 % significance level. Although the performance in *membrane* proteins was the worst, it needs to be noted that this set consisted of only a few proteins and should be treated with caution.

### Case Study Validation: KcsA Ion Channel

The case study of single potassium channel (KcsA) was conducted. The negative and positive knowledge in contact maps and an influence of reduced contact maps on the quality of models were tested.

#### Negative and Positive Knowledge in Contact Maps

Reconstruction of KcsA structure based on the complete contact map produces high quality models. Since the use of FT-COMAR step in the proposed reconstruction pipeline involves randomization, all produced models differ. Over 400 structures were generated. The average general RMSD value calculated for all complete-CMAP structures equaled 2.40 Å, which is in the resolution range of X-ray crystallography experimental structures. On the other hand, the results acquired for model-structures generated with positive-only-CMAPs were much worse (Table 2).

**Table 2:** Comparison between quality assessment of structures recovered from contact maps with complete knowledge and positive-only knowledge

	Complete-CMAP	Positive-only-CMAP
General RMSD [Å]	2.40 ± 0.14	6.43 ± 0.53
Filter residues RMSD [Å]	1.84 ± 0.39	3.95 ± 0.63
Structure diameter [Å]	41.92 ± 0.29	28.49 ± 4.57

**Fig. 6:**
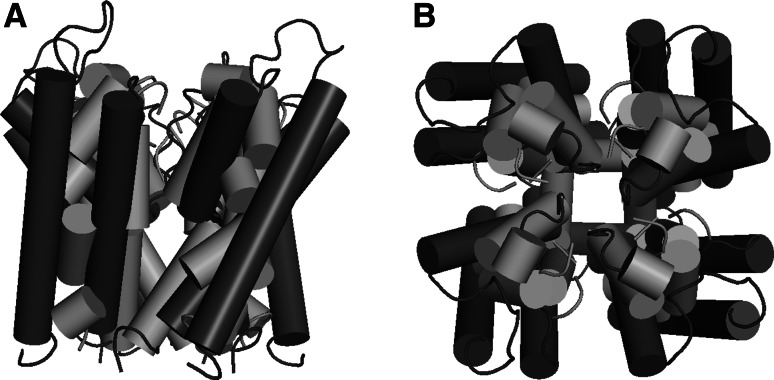
**a** Side view and **b** extracellular top view of aligned exemplary structures of KcsA. Models were reconstructed based on complete-CMAPs (*blue*) and positive-only-CMAPs (*red*) (Color figure online)

In Fig. 6, an exemplary alignment of two KcsA structures is presented. Secondary structures and their arrangement were correctly reconstructed in all structures based on complete positive and negative contact knowledge, i.e., complete-CMAPs (Fig. 6, blue). The lack of non-contacts in the map during generation of the second ensemble of models caused the protocol to produce very densely packed structures. The diameter of positive-only-CMAP structures, which was measured as the distance between two ALA-50 residues from two opposite chains, was lower than the diameter of complete-CMAP structures by over 10 Å (Table 2).

Although the general arrangement of alpha-helices is correct (Fig. 6, red) the whole geometry of models is unnatural, and the helices are broken. The only well reconstructed part of the channel is the selectivity filter, with average RMSD of 3.95 Å. In both groups of model-structures, the selectivity filter has a higher quality than the remaining parts of the structure.

#### Reduced Contact Maps

In order to find the minimal level of information required for reasonable reconstruction of models, the complete-CMAP was reduced to 90, 70, 50, and 30 % of contacts and non-contacts. Our results showed that at least 30 % of contacts are needed to obtain a structure of a reasonable quality. In another study, by Sathyapriya et al. 2009, the algorithm proposed for effectively reducing contact information indicated that only 8 % of contacts are needed for the structure reconstruction. A similar level was reported as sufficient by Kim et al. 2014, who used contacts provided by CASP10 organizers in contact-assisted CASP category (Taylor et al. 2014). In those studies, however, specially selected contacts were used for producing structures of good quality. In our study a random reduction of the contact map was carried out (see “Methods” section). Such an approach better mimics experimentally or computationally acquired contact site data since it does not require any special investigation of native structures, which are not available in real life situations.

**Fig. 7:**
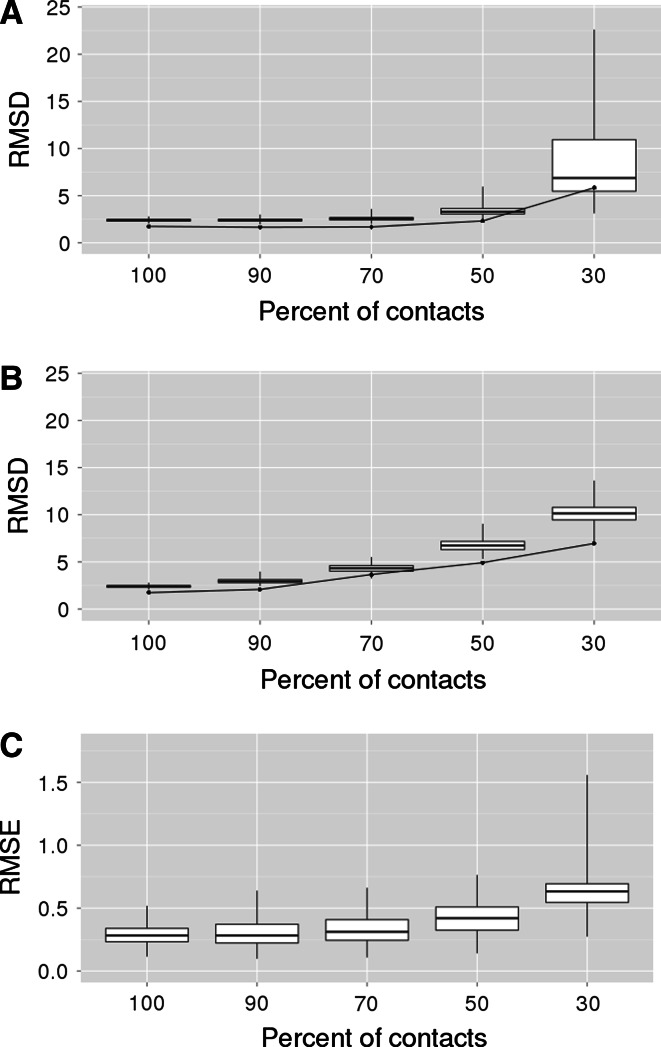
Quality of the reconstructed structures. The boxplots of the RMSD values in structures reconstructed based on reduced **a** error-free and **b** erroneous CMAPs. **c** The boxplot of the potential profile RMSE values in structures reconstructed based on reduced error-free CMAPs. Box borders denote QI and QIII quintiles, the median is marked with the *thick line*. Box whiskers denote 1.5 × InterQuartileRange. The *red line* in (**a**) and (**b**) marks the median RMSD in the selectivity filter (Color figure online)

The quality of produced models in 90 and 70 %-based model sets did not differ significantly from the ensemble of models reconstructed based on the complete-CMAP (Fig. 7a) and equaled approximately 2.5 Å. At the level of 50 % CMAP, the RMSDs became greater, also the spread of model quality increased. Only a small fraction of models, which were built based on 30 % of contact information could be useful, acquired satisfactory accuracy. However, if a good model quality assessment procedure was used, 30 % of knowledge would still be enough to generate structural models.

The ion channels need to have specific electrostatic potential distribution inside the pore to keep their functional properties. We decided to investigate if the RMSD value is a sufficient indicator for the quality assessment of ionic channel models. Hence we studied the relationship between RMSD values and similarity of electrostatic potential profiles along the pore axis.

**Fig. 8:**
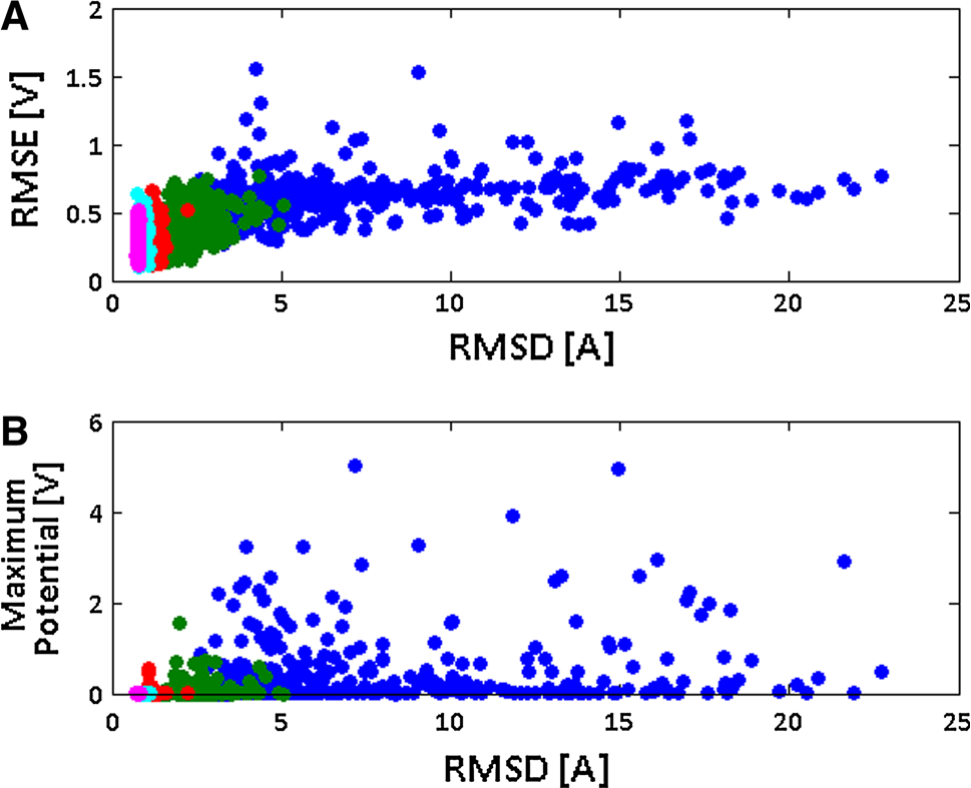
**a** The scatterplot of the potential profile RMSE values and the RMSD values. **b** The scatterplot of the maximum potential values and the RMSD values. The *circles* color depend on CMAPs completeness: 30 % reduced-CMAP (*blue*), 50 % reduced-CMAP (*dark green*), 70 % reduced-CMAP (*red*), 90 % reduced-CMAP (*cyan*), and complete-CMAPs (*magenta*) (Color figure online)

The RMSD and RMSE values of the models were correlated—*τ* Kendall correlation was 0.42, and *p*-value was 9.86 × 10^−145^ (Fig. 8a). The RMSE median differences between analyzed groups of models were statistically significant with the exception of the difference between complete-CMAP and 90 % reduced-CMAP difference (Fig. 7c). In case of RMSD medians, the differences between complete-CMAP, 90 and 70 % reduced-CMAP were not significant (Fig. 7a). The highest dispersion of the RMSE was observed for 30 % reduced-CMAP models (Figs. 7c, 8a, blue points), which is consistent with high spread of RMSD in this group of models (Fig. 7a). Similarly to RMSD, the boxplots of RMSE show the relation between the level of CMAP reduction and the quality of models. However, in case of RMSE, the quality differences are more evident, which suggests that the function dependent parameter—RMSE is more sensitive to quality changes than RMSD. In the group of 50 % reduced-CMAP models (Fig. 8a, green points) the dispersion of the RMSE values is greater than the dispersion of RMSD values. In groups of models based on more complete-CMAPs (Fig. 8a, red, cyan and magenta points, respectively, for 70, 90 % reduced-CMAP, and complete-CMAP) the differences between dispersions of the RMSEs and RMSDs are greater.

The electrostatic parameter, which strongly differentiated models, was the deviation of the maximum potential (Fig. 8b). The maximum potential value of the native KcsA structure equaled *Fmax* = 2.3 mV. The *τ* Kendall correlation between the RMSD and the *Fmax* deviation was 0.38 and p-value 8.51 × 10^−121^, but even in the group of models with high RMSD (between 5 and 15 Å) there were models with low *Fmax* deviation and among good structural models (RMSD lower than 5 Å) we found models with high *Fmax* deviation. The relationship between *Fmin* deviation and RMSD (figure not shown) was weaker than in case of RMSE and *Fmax*—the *τ* Kendall correlation was 0.24 and p-value 2.31 × 10^−50^. Even so, we observed differences between 30 % reduced-CMAP, 50 % reduced-CMAP and the remaining models. The *Fmin* deviations of complete-CMAP, 90 % reduced-CMAP and 70 % reduced-CAMP were very similar and ranged between 0 and 0.7, while for 50 % reduced-CMAP models they were greater. For instance, there were many models with *Fmin* deviation of about 1. In 30 % reduced-CMAP, there were four models which had the *Fmin* deviation even greater than 1. The *zmin* deviations acquired for the models could be described similarly to *Fmin*, i.e., while for complete, 90 and 70 % reduced-CMAP models the deviations were quite low, for 50 and 30 % reduced-CMAP *zmin* were much higher (data not shown).

The studies of RMSD and electrostatic potentials of KcsA showed that the majority of models reconstructed from complete, 90 and 70 % reduced-CMAPs were models of good quality. In those structures, low RMSD and correct electrostatic potential profiles were acquired. On the other hand, a number of KcsA models obtained from 50 % reduced-CMAPs and most models obtained from 30 % reduced-CMAP differed significantly from the native structure in terms of structure and electrostatic parameters. In most cases acquired electrostatic potentials were deformed with incorrectly located minima.

The conducted experiment was an idealized situation, since an assumption was made that the contact maps used were error-free. In real-life situations, this is never the case since the specificity of top state-of-the-art contact predictors varies around 0.3 (Monastyrskyy et al. 2011). Therefore, we also performed an experiment that mimics these conditions better.

Four sets of erroneous-CMAP KcsA structures were generated, i.e. 90, 70, 50, and 30 %. It was shown that the quality of models decayed rapidly as the balance between correct and erroneous contacts lowered (Fig. 7b). In a real-life situation, one needs to correctly predict at least 70 % of contacts to produce RMSD < 5 Å model-structures, which means that a significant improvement in the reliability of contact predictors is needed.

#### Local Quality of Structural Models Varies

In order to get an insight into the local quality of reconstructed models of KcsA, the full-atom RMSD of each amino acid residue was calculated. Based on that, local RMSDs were calculated. In the previous section, it was shown that in 90 and 70 % reduced-CMAP models the quality is almost exactly the same as in the complete-CMAP structures, while in the 50 % CMAP-based in many cases it is only slightly lower. Therefore, the local structure quality was investigated only in the complete-CMAP and 30 % reduced-CMAP structures.

**Fig. 9:**
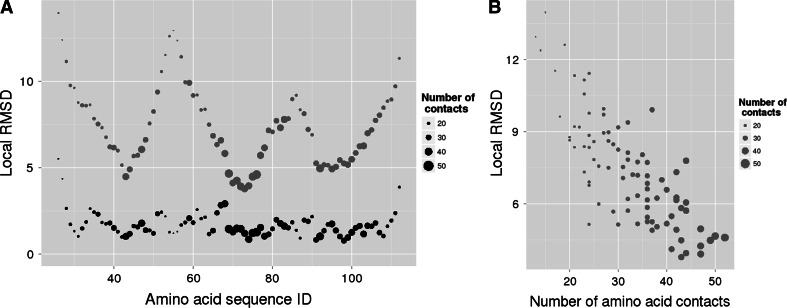
**a** The distribution of the local RMSD values along the amino acid sequence in complete-CMAP (*black*) and 30 % reduced-CMAP (*red*) models of KcsA. **b** Correlation between local RMSD in 30 % CMAP-based structures of KcsA and the number of contacts in which amino acids participate. *Point sizes* denote number of contacts amino acids participate in (Color figure online)

The averaged local RMSD values in the complete-CMAP models are distributed uniformly along the whole amino acid sequence (Fig. 9a, black), with the exception of terminal residues. There are only slight fluctuations and the structures are generally built correctly.

**Fig. 10:**
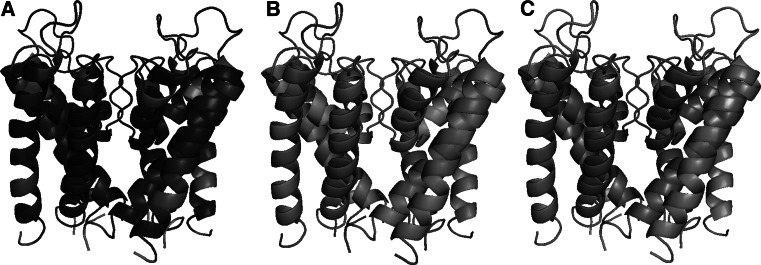
The local RMSD of **a** complete-CMAP and **b** 30 % CMAP-based structures mapped onto KcsA structure correlate well with **c** the mapping of the number of each amino acid contacts

Local quality in 30 % reduced-CMAP models varies significantly between different regions of the sequence. There are three clear segments of sequence where the local RMSDs are relatively low, i.e., between residues 39–47, 67–78, 91–105 (Fig. 9a, red). These segments are also associated with higher numbers of contacts created by amino acids (Fig. 9 point sizes). Figure 9b shows that number of contacts and local RMSDs are correlated. Local RMSDs were mapped onto 3D structure of the KcsA (Fig. 10a, b). The regions associated with high local qualities are located centrally in the protein structure, while peripheral parts of the structure, such as extra membrane loops or helix endings are poorly modeled.

RMSD and number of contact mappings onto the KcsA structure presented in Fig. 10 clearly show that regions of low RMSD (Fig. 10a, b blue/light green) overlap with regions occupied by amino acids with a high number of contacts (Fig. 10c, red).

The correlation observed between the number of contacts and local prediction accuracy is not surprising because contacts define constraints that need to be satisfied by the three-dimensional arrangement of amino acids. If an amino acid is involved in a high number of contacts and some of them are removed by a CMAP reduction procedure or missed by a contact prediction method, then the amino acid will be still properly localized in the 3D structure. This is because the information regarding the 3D position will be retained in the remaining contacts. Conversely, the 3D position of an amino acid that is involved in just a few contacts may be ambiguous, since many positions may satisfy constraints that are put on the amino acid. Therefore any method of protein structure prediction, based solely on the CMAP information, will be subject to this limitation. Furthermore, in order to get good quality of structures, it is important to secure a certain level of contact site information in all parts of the structure, which may be more difficult in protein peripheral parts.

## Conclusions

In the study, an automated protocol for protein structure reconstruction based on protein residue–residue contacts was proposed. Validation of the pipeline was performed using a set of randomly selected 205 diverse protein domain structures, which were downloaded from the SCOP-ASTRAL database. This was followed by a thorough case study of a single potassium ion channel (KcsA).

In the first step of the validation, the average RMSDs acquired for many investigated proteins were quite low, between 3 and 5 Å, however the overall average equaled 6.45 Å, which was higher than the value reported by the authors of FT-COMAR. The study showed that there was a relation between the accuracy of produced models and the contact density of a protein. The protocol was much more successful in cases where more than 10 residue–residue contacts per amino acid appeared. In those cases RMSD values fluctuated around 4 Å. The analysis did not prove that protein length influences the accuracy of predictions.

The highest prediction accuracy (the lowest RMSD) was reported for proteins from *all-beta*, *alpha* *+* *beta* and *small* SCOP-ASTRAL protein classes. We propose that these involve proteins with the favorable packing characteristics.

In the KcsA channel case study, several procedures were used to generate CMAP containing different types and portions of information, i.e., complete and positive-only contact maps, reduced and erroneous contact maps. It was shown that for a proper reconstruction of models, both types of knowledge, positive (regarding contacting residues) and negative (related to non-contacting amino acids) are required. If this is satisfied, then even 30 % of contact information is enough to produce structural models with RMSD below 5 Å. Although similar studies done by other authors using different algorithms (Sathyapriya et al. 2009, Kim et al. 2014) showed that only 8 % of contacts are needed for the structure reconstruction, this is true only if the contacts are selected non-randomly and the full knowledge of protein structure is applied for this selection. Our study also proves that introduction of errors in the contact map significantly lowers the quality of produced structures and at least 70 % of contact site information is needed to acquire reasonable models. Therefore, if such a contact-based approach is to be applicable in real-life situations, it is of great importance to assure a low level of the false positive rate of provided contact predictions.

The case study reveals that structure prediction accuracy (RMSD) and electrostatic properties of models are correlated. RMSE of electrostatic potential profile in the channels axis and the *Fmax* of the profile were the most correlated electrostatic parameters. These parameters could be used as indicators of the model quality.

Investigation of structures local quality revealed that some regions of models were predicted with higher accuracy. These regions overlapped with regions of high contact density. It was confirmed that the reconstruction quality is directly related to the number of contacts in which amino acid is involved. This dependence will affect all contact-based approaches to protein structure predictions.

## References

[CR1] Arnold K, Bordoli L, Kopp J, Schwede T (2006). The SWISS-MODEL Workspace: A web-based environment for protein structure homology modelling. Bioinformatics.

[CR2] Baker NA, Sept D, Joseph S, Holst MJ, McCammon JA (2001). Electrostatics of nanosystems: application to microtubules and the ribosome. Proc. Natl. Acad. Sci..

[CR3] Berman HM, Westbrook J, Feng Z, Gilliland G, Bhat TN, Weissig H, Shindyalov IN, Bourne PE (2000). The Protein Data Bank. Nucl. Acids Res.

[CR4] Camproux A, Gautier R, Tuffery P (2004). A Hidden Markov Model Derived Structural Alphabet for Proteins. J. Mol. Biol..

[CR5] Duarte JM, Sathyapriya R, Stehr H, Filippis I, Lappe M (2010). Optimal contact definition for reconstruction of contact maps. BMC Bioinformatics.

[CR6] Duarte JM, Sathyapriya R, Stehr H, Filippis I, Lappe M (2010b) RECONSTRUCT - Protein contact map reconstruction using the TINKER package, http://www.bioinformatics.org/owl/reconstruct/index.html

[CR7] Dyrka W, Augousti AT, Kotulska M (2008). Ion flux through membrane channels–an enhanced algorithm for the Poisson-Nernst-Planck model. J Comput Chem.

[CR8] Dyrka W, Bartuzel MM, Kotulska M (2013). Optimization of 3D Poisson-Nernst-Planck model for fast evaluation of diverse protein channels. Proteins.

[CR9] Ebejer JP, Hill JR, Kelm S, Shi J, Deane CM (2013). Memoir: template-based structure prediction for membrane proteins. Nucleic Acids Res.

[CR10] Havel TF, Kuntz ID, Crippen GM (1983). The combinatorial distance geometry method for the calculation of molecular conformation. I. A new approach to an old problem. J Theor Biol.

[CR11] Ho HK, Kuiper MJ, Kotagiri R (2008). PConPy–a Python module for generating 2D protein maps. Bioinformatics.

[CR12] Hopf TA, Colwell LJ, Sheridan R, Rost B, Sander C, Marks DS (2012). Three-dimensional structures of membrane proteins from genomic sequencing. Cell.

[CR13] Jones DT, Buchan DW, Cozzetto D, Pontil M (2012). PSICOV: precise structural contact prediction using sparse inverse covariance estimation on large multiple sequence alignments. Bioinformatics.

[CR14] Källberg M, Wang H, Wang S, Peng J, Wang Z, Lu HXuJ (2012). Template-based protein structure modeling using the RaptorX web server. Nature Protocols.

[CR15] Kelley LA, Sternberg MJE (2009). Protein structure prediction on the web: a case study using the Phyre server. Nat Protoc.

[CR16] Kim DE, Dimaio F, Yu-Ruei Wang R, Song Y, Baker D (2014). One contact for every twelve residues allows robust and accurate topology-level protein structure modeling. Proteins.

[CR17] Kozma D, Simon I, Tusnády GE (2012) PDBTM: Protein Data Bank of transmembrane proteins after 8 years. Nucleic Acids Research 33 Database Issue: D275-D278 10.1093/nar/gks1169 PMC3531219 23203988

[CR18] Krivov GG, Shapovalov MV, Dunbrack RL (2009). Improved prediction of protein side–chain conformations with SCWRL4. Proteins.

[CR19] Kryshtafovych A, Fidelis K, Moult J (2013) CASP10 results compared to those of previous CASP experiments. Proteins, “Accepted Article”, Proteins 82 Suppl 2:164-74. doi: 10.1002/prot.24448 10.1002/prot.24448 PMC4180100 24150928

[CR20] Lomize MA, Lomize AL, Pogozheva ID, Mosberg HI (2006). OPM: Orientations of Proteins in Membranes database. Bioinformatics.

[CR21] Marks DS, Colwell LJ, Sheridan R, Hopf TA, Pagnani A (2011). Protein 3D Structure Computed from Evolutionary Sequence Variation. PLoS ONE.

[CR22] Maupetit J, Gautier R, Tuffery P (2006) SABBAC: online Structural Alphabet-based protein Back Bone reconstruction from Alpha-Carbon trace. Nucl Acids Res 34 (Web Server issue):W147–W151 10.1093/nar/gkl289 PMC1538914 16844979

[CR23] Monastyrskyy B, Fidelis K, Tramontano A, Kryshtafovych A (2011). Evaluation of residue–residue contact predictions in CASP9. Proteins.

[CR24] Nugent T, Jones DT (2012). Accurate de novo structure prediction of large transmembrane protein domains using fragment-assembly and correlated mutation analysis. Proc Natl Acad Sci U S A.

[CR25] Jafri MS, Kotulska M (2006). Modeling the mechanism of metabolic oscillations in ischemic cardiac myocytes. J Theor Biol.

[CR26] Sathyapriya R, Duarte JM, Stehr H, Filippis I, Lappe M (2009). Defining an Essence of Structure Determining Residue Contacts in Proteins. PLoS Comput Biol.

[CR27] Söding J, Biegert A, Lupas AN (2005) The HHpred interactive server for protein homology detection and structure prediction. Nucleic Acids Res. 33(Web Server issue):W244–8 10.1093/nar/gki408 PMC1160169 15980461

[CR28] Taylor TJ, Bai H, Tai CH, Lee B (2014). Assessment of CASP10 contact-assisted predictions. Proteins.

[CR31] Taylor WR, Jones DT, Sadowski MI (2012). Protein topology from predicted residue contacts. Protein Sci.

[CR29] Tuffery P, Guyon F, Derreumaux P (2005). Improved Greedy Algorithm for Protein Structure Reconstruction. J Comp Chem.

[CR30] Vassura M, Margara L, Di Lena P, Medri F, Fariselli P, Casadio R (2008). FT-COMAR: fault tolerant three-dimensional structure reconstruction from protein contact maps. Bioinformatics.

[CR32] Vendruscolo M, Kussell E, Domany E (1997). Recovery of protein structure from contact maps. Fold Des.

